# Genetic approaches to metabolic bone diseases

**DOI:** 10.1111/bcp.13803

**Published:** 2018-11-28

**Authors:** Fadil M. Hannan, Paul J. Newey, Michael P. Whyte, Rajesh V. Thakker

**Affiliations:** ^1^ Academic Endocrine Unit, Radcliffe Department of Medicine, University of Oxford Oxford UK; ^2^ Department of Musculoskeletal Biology, Institute of Ageing and Chronic Disease University of Liverpool Liverpool UK; ^3^ Division of Molecular & Clinical Medicine, Ninewells Hospital & Medical School University of Dundee UK; ^4^ Center for Metabolic Bone Disease and Molecular Research Shriners Hospital for Children St. Louis MO 63110 USA; ^5^ Division of Bone and Mineral Diseases, Department of Internal Medicine Washington University School of Medicine at Barnes‐Jewish Hospital St. Louis MO 63110 USA

**Keywords:** genetic diseases, genetics and pharmacogenetics, molecular biology, osteoporosis, rheumatology

## Abstract

Metabolic bone diseases comprise a diverse group of disorders characterized by alterations in skeletal homeostasis, and are often associated with abnormal circulating concentrations of calcium, phosphate or vitamin D metabolites. These diseases commonly have a genetic basis and represent either a monogenic disorder due to a germline or somatic single gene mutation, or an oligogenic or polygenic disorder that involves variants in more than one gene. Germline single gene mutations causing Mendelian diseases typically have a high penetrance, whereas the genetic variations causing oligogenic or polygenic disorders are each associated with smaller effects with additional contributions from environmental factors. Recognition of familial monogenic disorders is of clinical importance to facilitate timely investigations and management of the patient and any affected relatives. The diagnosis of monogenic metabolic bone disease requires careful clinical evaluation of the large diversity of symptoms and signs associated with these disorders. Thus, the clinician must pursue a systematic approach beginning with a detailed history and physical examination, followed by appropriate laboratory and skeletal imaging evaluations. Finally, the clinician must understand the increasing number and complexity of molecular genetic tests available to ensure their appropriate use and interpretation.

## Introduction

Metabolic bone diseases represent a diverse group of skeletal conditions characterized by alterations in bone cell activity, bone matrix proteins or systemic mineral homeostasis (Table 1) 1, 2. Many metabolic bone diseases have a genetic basis, which may be a germline single gene abnormality (i.e. a monogenic or Mendelian disorder), a somatic single gene defect (i.e. a post‐zygotic mosaic disorder) or involve several genetic variants (i.e. oligogenic or polygenic disorders) 3. Genetic mutations causing Mendelian diseases usually have a large effect (i.e. penetrance), whereas oligogenic or polygenic disorders are associated with several genetic variations, each of which may have smaller effects with greater or smaller contributions from environmental factors (i.e. multifactorial disorders) 3. Whilst many monogenic disorders result from rare mutations affecting the coding sequence of the responsible gene, the majority of common genetic variants identified in association with polygenic traits are located in non‐coding regions, usually in proximity to candidate genes implicated in the respective disorders 4. Furthermore, there is substantial overlap between the genes responsible for monogenic skeletal diseases and those contributing to polygenic bone phenotypes. The elucidation of these loci has provided insights into the molecular pathogenesis of skeletal disease, and highlighted novel therapeutic targets 5, 6, 7. This review discusses the genetics of metabolic bone diseases, and outlines the clinical and genetic approach to evaluating these disorders.

**Table 1:** Examples of monogenic metabolic bone disorders, modes of inheritance and genetic aetiology

Mode of inheritance/Disease	Gene(s)	Chromosomal location	References
***Autosomal dominant***			
**Osteogenesis imperfecta (OI), types I‐IV**	*COL1A1, COL1A2*	17q21.33, 7q21.3	20
**Osteogenesis imperfecta (OI), type V**	*IFITM5*	11p15.5	23, 24
**Autosomal dominant hypophosphataemic rickets**	*FGF23*	12p13.32	25
**Autosomal dominant high bone mass, type 1**	*LRP5*	11q13.2	47
**Autosomal dominant high bone mass, type 2**	*LRP6*	12p13.2	48
**Early‐onset osteoporosis**	*WNT1*	12q13.12	19
**Familial hypocalciuric hypercalcaemia (FHH), types 1–3**	*CASR, GNA11, AP2S1*	3q21.1, 19p13.3, 19q13.3	31, 32, 33
**Autosomal dominant hypocalcaemia (ADH), types 1–2**	*CASR, GNA11*	3q21.1, 19p13.3	32, 37
**Familial expansile osteolysis**	*TNFRSF11A*	18q21.33	34, 35
**Hypophosphatasia**	*TNSALP/ALPL*	1p36.12	36
**Vitamin D‐dependent rickets, type 3**	*CYP3A4*	7q22.1	88
**Pseudohypoparathyroidism, type 1a (PHP1a)** a	*GNAS*	20q13.3	39
**Pseudopseudohypoparathyroidism (PPHP)** a	*GNAS*	20q13.3	39
**Pseudohypoparathyroidism, type 1b (PHP1b)** a	*GNAS, NESP55, STX16*	20q13.3	39
***Autosomal recessive***			
**Osteogenesis imperfecta (OI), type VI**	*SERPINF1*	17p13.3	106
**Osteogenesis imperfecta (OI), type VII**	*CRTAP*	3p22.3	21
**Osteogenesis imperfecta (OI), type VIII**	*P3H1/LEPRE1*	1p34.2	107
**Osteogenesis imperfecta (OI), type XV**	*WNT1*	12q13.12	19
**Hypophosphatasia**	*TNSALP/ALPL*	1p36.12	36
**Neonatal severe hyperparathyroidism (NSHPT)**	*CASR*	3q21.1	31
**Vitamin D‐dependent rickets, type 1**	*CYP27B1*	12q14.1	10
**Vitamin D‐dependent rickets, type 2**	*VDR*	12q13.11	10
**Autosomal recessive hypophosphataemic rickets**	*DMP1, ENPP1*	4q22.1, 6q23.2	27, 28
**Hereditary hypophosphataemic rickets with hypercalciuria**	*SLC34A3*	9q34.3	29, 30
**Osteoporosis‐pseudoglioma syndrome**	*LRP5*	11q13.2	46
**Sclerosteosis, type 1**	*SOST*	17q21.31	49
**Sclerosteosis, type 2**	*LRP4*	11p11.2	50
**Pyle's disease**	*SFRP4*	7p14.1	54
**Juvenile Paget disease**	*TNFRSF11B*	8q24.12	108
***X‐linked dominant***			
**X‐linked hypophosphatemic (XLH) rickets**	*PHEX*	Xp22.11	26
***X‐linked recessive***			
**X‐linked osteoporosis**	*PLS3*	Xq23	18
**Dent disease, type 1**	*CLCN5*	Xp11.23	11
***Mitochondrial***			
**Mitochondrial encephalomyopathy with lactic acidosis and stoke‐like episodes (MELAS)**	Mitochondrial genome	‐	13
**Kearns‐Sayre syndrome**	Mitochondrial genome	‐	14
***Mosaicism***			
**McCune‐Albright syndrome (polyostotic fibrous dysplasia)** a	*GNAS*	20q13.3	15
**Osteogenesis imperfecta (OI)** b	*COL1A1*/*COL1A2*	17q21.33, 7q21.3	

a Parentally imprinted

b Autosomal disorder manifesting as post‐zygotic somatic mosaicism in the developing fetus, or arising from germline mosaicism in an apparently unaffected parent

## Genetics of metabolic bone diseases

### Inheritance

Metabolic bone diseases may be caused by single‐gene mutations or represent digenic or complex polygenic traits 1, 3, 8. Inheritance of monogenic diseases occurs as one of six traits: autosomal dominant [e.g. familial hypocalciuric hypercalcaemia (FHH) due to mutations of the calcium‐sensing receptor (CaS receptor) signalling pathway 9]; autosomal recessive [e.g. vitamin D‐dependent rickets types 1 and 2 from mutations of the renal 1α‐hydroxylase (*CYP27B1*) and vitamin D receptor (*VDR*) genes, respectively 10]; X‐linked recessive [e.g. Dent's disease involving chloride channel 5 (CLC‐5) 11]; X‐linked dominant [e.g. X‐linked hypophosphataemic (XLH) rickets from mutations of a phosphate endopeptidase on the X chromosome (*PHEX*) gene 10]; Y‐linked (e.g. azoospermia and oligospermia) 12; and non‐Mendelian mitochondrial defects [e.g. hypoparathyroidism in Kearns‐Sayre syndrome and mitochondrial encephalopathy, lactic acidosis and stroke‐like episodes (MELAS) syndrome] 13, 14. Monogenic metabolic bone diseases may also be caused by sporadic postzygotic mosaicism [e.g. McCune‐Albright syndrome (MAS)] (Table 1) 15. Digenic inheritance has been reported in a family with hereditary hypophosphataemic rickets with hypercalciuria (HHRH), who harbour heterozygous mutations of the *SLC34A1* and *SLC34A3* genes, encoding the renal sodium‐phosphate co‐transporters type 2a and 2c, respectively 8. The major metabolic bone disorder representing a complex polygenic trait is osteoporosis, and more than 200 loci have been associated with this common disorder 16, 17. However, the majority of loci for osteoporosis likely remain to be elucidated. Osteoporosis may rarely occur as a monogenic condition, e.g. X‐linked osteoporosis due to mutations of the Plastin 3 (*PLS3*) gene 18, or early‐onset osteoporosis due to heterozygous mutations of the Wnt family member 1 (*WNT1*) gene (Table 1) 19.

### Genetic heterogeneity

Many phenotypically similar metabolic bone disorders are caused by mutations in a variety of different genes. For example, 85–90% of osteogenesis imperfecta (OI) cases are due to mutations in the genes encoding type 1 collagen (i.e. *COL1A1* and *COL1A2*) 20, with the remaining 10–15% of OI cases being caused by mutations affecting genes involved in post‐translational processing of collagen [e.g. cartilage‐associated protein (*CRTAP*)] 21, osteoblast differentiation and function (e.g. *WNT1*) 19, 22, or bone mineralization [e.g. interferon‐induced transmembrane protein 5 (*IFITM5*)] (Table 1) 23, 24. Similarly, hypophosphataemic rickets may be caused by mutations of genes encoding phosphatonins like fibroblast growth factor‐23 (FGF‐23), or osteoblast and osteocyte proteins that mediate the expression and secretion of FGF‐23 [e.g. *PHEX*, dentin matrix protein 1 (*DMP1*), and ectonucleotide pyrophosphatase/phosphodiesterase 1 (*ENPP1*)] 25, 26, 27, 28, or by mutations affecting renal sodium phosphate co‐transporters (e.g. *SLC34A3)* (Table 1) 29, 30. In addition, FHH, which is a disorder of extracellular calcium homeostasis, has been shown to comprise three types, which are caused by germline loss‐of‐function mutations affecting the CaS receptor, G‐protein subunit‐α11 (Gα_11_), and adaptor‐related protein complex‐2 σ‐subunit (AP2σ)*,* respectively (Table 1) 31, 32, 33.

Mutations within a single gene may give rise to seemingly distinctive skeletal phenotypes [(e.g. familial expansile osteolysis (FEO), expansile skeletal hyperphosphatasia (ESH) and early‐onset familial Paget's disease of bone (PDB)], which are rapid remodelling skeletal disorders arising from mutations in the signal peptide of receptor activator of NF‐κB (RANK) 34, 35. In some metabolic bone diseases, the severity may be determined by mutant allele dosage and whether a mutation is carried in the heterozygous or homozygous state. For example, the severe perinatal and infantile forms of hypophosphatasia, an inborn‐error‐of‐metabolism characterized by alkaline phosphatase (ALP) deficiency, are inherited in an autosomal recessive manner, whilst later‐onset and milder forms are typically inherited in an autosomal dominant fashion (Table 1) 36. Moreover, some disorders of mineral metabolism are caused by loss‐ or gain‐of‐function mutations affecting the same gene. Thus, loss‐of‐function CaS receptor mutations cause FHH or neonatal severe hyperparathyroidism (NSHPT), whereas gain‐of‐function CaS receptor mutations cause autosomal dominant hypocalcaemia (ADH) or Bartter syndrome type V 31, 37, 38. Furthermore, parental imprinting, which results in non‐Mendelian inheritance of a monogenic disorder, may influence the phenotypic consequences of a specific mutation. For example, maternally‐inherited inactivating coding‐region mutations of G‐protein subunit αs (Gαs), which is encoded by the *GNAS* gene, cause pseudohypoparathyroidism type 1a (PHP1a), which is characterized by PTH resistance together with Albright's hereditary osteodystrophy (AHO) 39; whereas, paternally‐inherited inactivating coding‐region *GNAS* mutations cause pseudopseudohypoparathyroidism (PPHP), which is characterized by AHO without PTH resistance (Table 1) 39. The phenotype of MAS, which is caused by somatic activating Gαs mutations, is also dependent on parental imprinting, with acromegaly occurring in MAS patients who harbour mutations affecting the maternal Gαs allele 40. Given this apparent genetic/phenotypic complexity despite genetic ‘homogeneity’, establishing the genetic cause can be challenging for the evaluation of patients and family members with bone and mineral disorders.

### Molecular insights from monogenic and polygenic diseases

**Figure 1:**
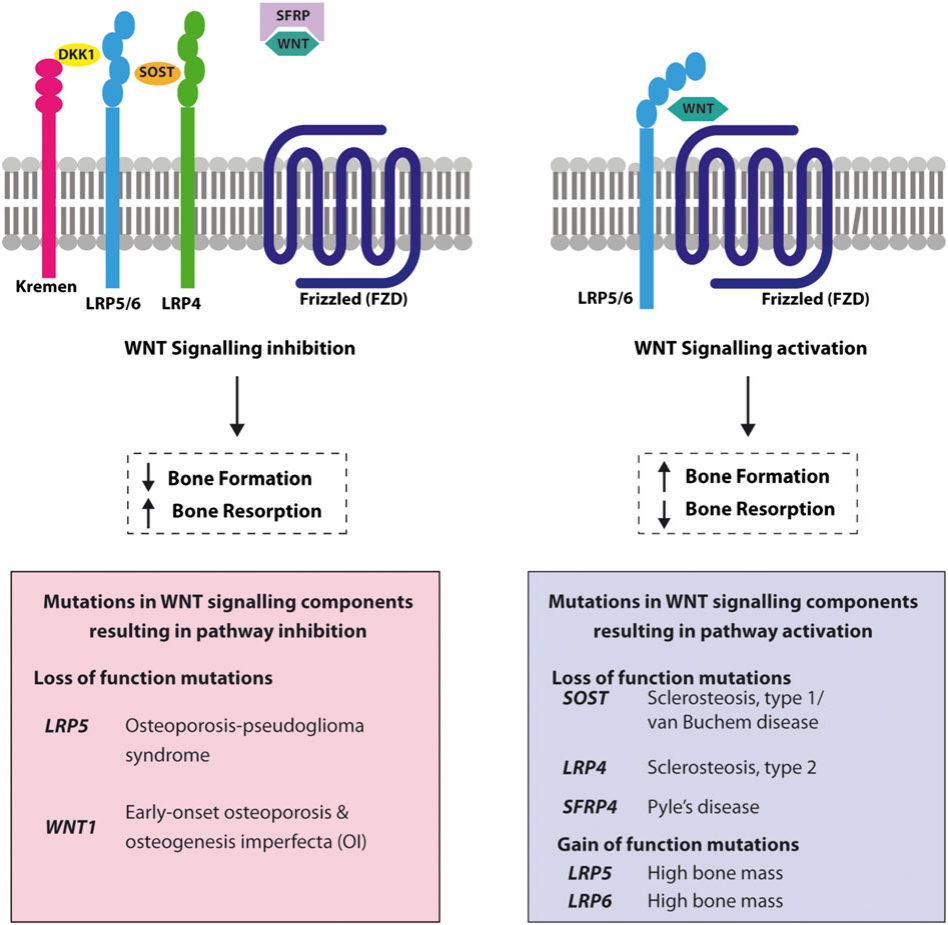
Schematic representation of Wnt signalling pathway components reported to be mutated in disorders of bone development and skeletal homeostasis. Activation of the canonical Wnt pathway increases bone mass, and this is mediated by the binding of extracellular Wnt ligands (dark green) to a transmembrane receptor complex comprising the Wnt co‐receptor LRP5 or LRP6 (LRP5/6, light blue) and a member of the frizzled (FZD) family (dark blue). In contrast, inhibition of the canonical Wnt pathway decreases bone mass 44, 45. This inhibition is mediated by extracellular factors such as sclerostin (SOST, orange) and Dickkopf‐related protein 1 (DKK1, yellow), which bind to the LRP5/6 co‐receptor thereby preventing activation by Wnt ligands, as well as recruiting inhibitory transmembrane proteins such as LRP4, which is a SOST‐interacting protein (light green), and the Kremen proteins (pink), which are high‐affinity DKK1 receptors that functionally cooperate with DKK1 to decrease Wnt signalling 109. Secreted‐frizzled‐related proteins (SFRPs, purple) also inhibit the canonical Wnt pathway by sequestering Wnt ligands. The importance of the canonical Wnt pathway for the regulation of bone mass has been highlighted by loss‐of‐function mutations affecting SOST and LRP4, and by gain‐of‐function mutations of LRP5 and LRP6, which lead to the disorder called high bone mass 47, 49, 51, 110; and also by loss‐of‐function mutations of LRP5 and the Wnt1 ligand, which lead to monogenic osteoporosis disorders 19, 46

Classical gene discovery approaches for monogenic disorders have involved studying affected kindreds for co‐segregation with polymorphic genetic markers to define the chromosomal location, followed by DNA sequence analysis of genes located within the candidate region 3. This approach has been superseded by whole‐exome and whole‐genome sequence analysis of affected patients or kindreds 41, 42. In contrast, the genetic investigation of complex polygenic disorders such as osteoporosis has utilized genome‐wide association studies (GWAS), which involve large populations of cases and controls 5, 6, 16, 17. Such studies typically involve direct or imputed genotyping of large numbers of common (e.g. minor allele frequency >5%) and infrequent (e.g. minor allele frequency 1–5%) single nucleotide polymorphisms/variants (SNPs/SNVs) to identify genetic loci enriched for the trait 3, 43. The genetic investigation of monogenic diseases has provided a fundamental understanding of the molecular regulation of bone mass and maintenance of skeletal microarchitecture. For example, studies of mutations affecting several Wnt pathway components have demonstrated that Wnt signalling plays a key anabolic role in the skeleton (Figure 1) 44, 45. Thus, autosomal‐recessive loss‐of‐function mutations of the *LRP5* gene, which encodes a key Wnt co‐receptor (Figure 1), result in osteoporosis‐pseudoglioma syndrome, which is characterized by severe juvenile osteoporosis and congenital or childhood‐onset blindness 46. In contrast, heterozygous activating mutations in *LRP5* 47 and *LRP6* 48, which encode the cognate co‐receptors LRP5 and LRP6, respectively, both lead to autosomal dominant high bone mass. Additionally, individuals with autosomal recessive loss‐of‐function mutations of the Wnt‐β‐catenin inhibitor sclerostin (*SOST*) manifest sclerosteosis, type 1, which is characterized by progressive bone overgrowth throughout life 49, 50; whilst patients harbouring a homozygous 52 kb deletion containing an enhancer element downstream of the *SOST* gene develop van Buchem disease, which has a similar but milder skeletal phenotype compared to sclerosteosis, type 1 51, 52. Moreover, bi‐allelic loss‐of‐function mutations of *WNT1* have been shown to cause an autosomal recessive form of OI, whilst heterozygous carriers of such *WNT1* missense mutations develop autosomal dominant early‐onset osteoporosis (Figure 1) 19, 53. Additionally, bi‐allelic truncating mutations in secreted frizzled‐related protein 4 (sFRP‐4) (Figure 1)*,* which encodes a soluble Wnt inhibitor, have been reported in patients with Pyle's disease, a disorder characterized by cortical bone thinning, limb deformity and fracture 54. These key roles for Wnt signalling in bone biology are supported by the findings from GWAS studies, which have identified that many Wnt pathway components (>15 genes), including *LRP5* and *SOST*, are candidate genes for bone mineral density (BMD) 16, 17, and that *WNT16* is a key determinant of cortical bone strength 55, 56.

### Application of genetic discoveries to the development of targeted therapies

A key aim of the genetic characterization of metabolic bone disorders has been to identify genes, molecules and pathways that may be targeted therapeutically. Thus, the identification of the bone cell OPG/RANKL/RANK/NF‐κB signalling pathway led to the development of the monoclonal antibody denosumab, which blocks RANK ligand (RANKL), thereby inhibiting osteoclast‐mediated bone resorption 5. Denosumab is now widely used for the treatment of osteoporosis as it significantly reduces fracture risk in women with postmenopausal osteoporosis 57. The multinational approval in 2015 of the bone‐targeted enzyme‐replacement biologic asfotase alfa to treat hypophosphatasia has emphasized the importance of determining the genetic and molecular basis for a metabolic bone disease 36. The identification that *PHEX* mutations cause FGF‐23 excess, which in turn is responsible for the phosphate wasting in XLH 58, 59, has led to the approval in 2018 of burosumab, which is an anti‐FGF‐23 monoclonal antibody, for the treatment of XLH rickets. Burosumab has been shown to improve serum phosphate concentrations and decrease the severity of rickets in children with XLH 60. Assessing treatment response according to the genetic aetiology has been investigated in patients with early‐onset low‐turnover osteoporosis due to *WNT1* or *PLS3* mutations who were shown to respond to teriparatide therapy 61. Now, several drugs in development are directed at the Wnt pathway. This includes anti‐sclerostin antibodies (e.g. romosozumab), which increase bone formation whilst inhibiting bone resorption 62. An evaluation of romosozumab in phase 3 clinical trials has shown that it is a potent bone anabolic agent for postmenopausal osteoporosis 63, 64.

## Clinical approach to the patient with a metabolic bone disease

### Medical history and physical examination

The diagnosis of genetic forms of metabolic bone diseases begins by acquiring information from the patient's medical history and physical examination 3. The ‘history of present illness’ provides critical clues concerning aetiology, pathogenesis and prognosis, as well as guiding diagnosis and therapy. Establishing whether the signs and symptoms have been lifelong or begun recently may prompt different diagnostic considerations and interventions. Thus, lifelong fractures which have occurred following minor trauma may suggest a diagnosis of OI 20, whereas the combined occurrence of fractures and renal calculi in early adulthood may potentially be a presenting feature of primary hyperparathyroidism caused by the multiple endocrine neoplasia (MEN) type 1 syndrome 65. Moreover, it is important to review prior medical records, radiographs and other investigations, such as the results of plasma and urinary biochemistry, to aid diagnosis and prognostication 3. Physical assessment should include: measurement of body proportions, limb lengths and head circumference; an examination of the spine for scoliosis or kyphosis; and joint hypermobility with a determination of the Beighton score 66. Physical examination can show a variety of findings for diagnosis, e.g. blue or grey sclerae found in OI; café‐au‐lait spots or other pigmentary cutaneous lesions that are associated with disorders of FGF‐23 excess such as MAS or the epidermal nevus syndrome; angiofibromas or collagenomas that may be associated with MEN type 1; premature loss of deciduous teeth that occurs in hypophosphatasia; hallux valgus which is found in fibrodysplasia ossificans progressiva; alopecia that occurs in vitamin D‐dependent rickets, type 2; brachydactyly which is found in PHP1a and PPHP; syndactyly that occurs in sclerosteosis types 1 and 2; torus palatinus which is found in disorders of high bone mass due to *LRP5* or *LRP6* mutations; or numerous surgical scars which may reveal a past medical history of surgical treatments to remove endocrine tumours associated with the MEN syndromes 10, 20, 36, 39, 48, 50, 65, 67, 68, 69. For some genetic bone diseases, a constellation of physical features indicates the category for diagnosis; e.g. rickets featuring craniotabes at birth and soon after a rachitic rosary (enlargement of the costochondral junctions) appearing during the first year of life 10. Childhood‐onset rickets causes bowed legs, short stature, flared wrists and ankles from metaphyseal widening 10. Knock‐knee deformities may occur instead of bowed legs if the rachitic disturbance occurs during the adolescent growth spurt 3. In adults, skeletal deformation originating from metabolic bone disorders in childhood can cause substantial morbidity. Bowing of the lower limbs predisposes to osteoarthritis, especially affecting the knees. Without a complete physical examination, these important problems may go unnoticed.

### Family history

Assessment of the family history is essential for establishing the mode of inheritance of monogenic metabolic bone diseases, and medical records from living or deceased affected family members may establish the diagnosis, guide prognostication, and indicate a safe and effective treatment 3. In autosomal dominant disease, the affected person often has one affected parent, and the disease occurs in both sexes and is transmitted by either the father or mother. In autosomal recessive diseases, which can affect both sexes, the proband is born to parents who are usually asymptomatic ‘carriers’ and sometimes related (i.e. consanguineous). In X‐linked recessive diseases, usually only males are affected, parents are unaffected yet the mother is an asymptomatic carrier, and there is no male‐to‐male transmission. In X‐linked dominant diseases, both males and females can be affected, although the females are often more mildly and variably affected than males, and 50% of offspring (girls and boys) from an affected woman will have the disease, and 100% of the daughters but 0% of sons of an affected man will have the disease. In Y‐linked diseases, only males are affected and, unless representing a sporadic case, they have an affected father (patrilineal inheritance) and all sons of an affected male will have the disease. Mitochondrial inherited disorders (non‐Mendelian) can affect both sexes. However, these disorders are only transmitted by an affected mother (matrilineal inheritance) in her egg mitochondrial DNA, and not through the paternal line in the sperm, as the small volume of sperm precludes them from contributing mitochondria to the zygote 3. These patterns of inheritance may be complicated by: non‐penetrance or variable expression in autosomal dominant disorders (e.g. in MEN1) 65; imprinting whereby expression of an autosomal dominant disorder is conditioned by whether it is maternally or paternally transmitted (e.g. PHP1a versus PPHP) 39; anticipation, whereby some dominant disorders become more severe (or have earlier onset) in successive generations; pseudo‐dominant inheritance of autosomal recessive disorders reflecting repeated consanguineous marriages in successive generations; and mosaicism in which an individual has two or more populations of cells with different genotypes because of post‐zygotic mutations during their development from a single fertilized egg (e.g. MAS). In the special circumstance of germline mosaicism within eggs or sperm arising from somatic mutation during gametogenesis, there may be confusion about the diagnosis and recurrence risk because of seemingly unaffected parents having multiple affected offspring that would be consistent with autosomal recessive inheritance, but actually reflects an autosomal dominant disorder (e.g. OI type II) 70. Hence, these inheritance patterns, which can help to diagnose a genetic disorder and identify individuals at risk, can come from a detailed family history 3.

### Clinical utility of genetic investigations

**Figure 2:**
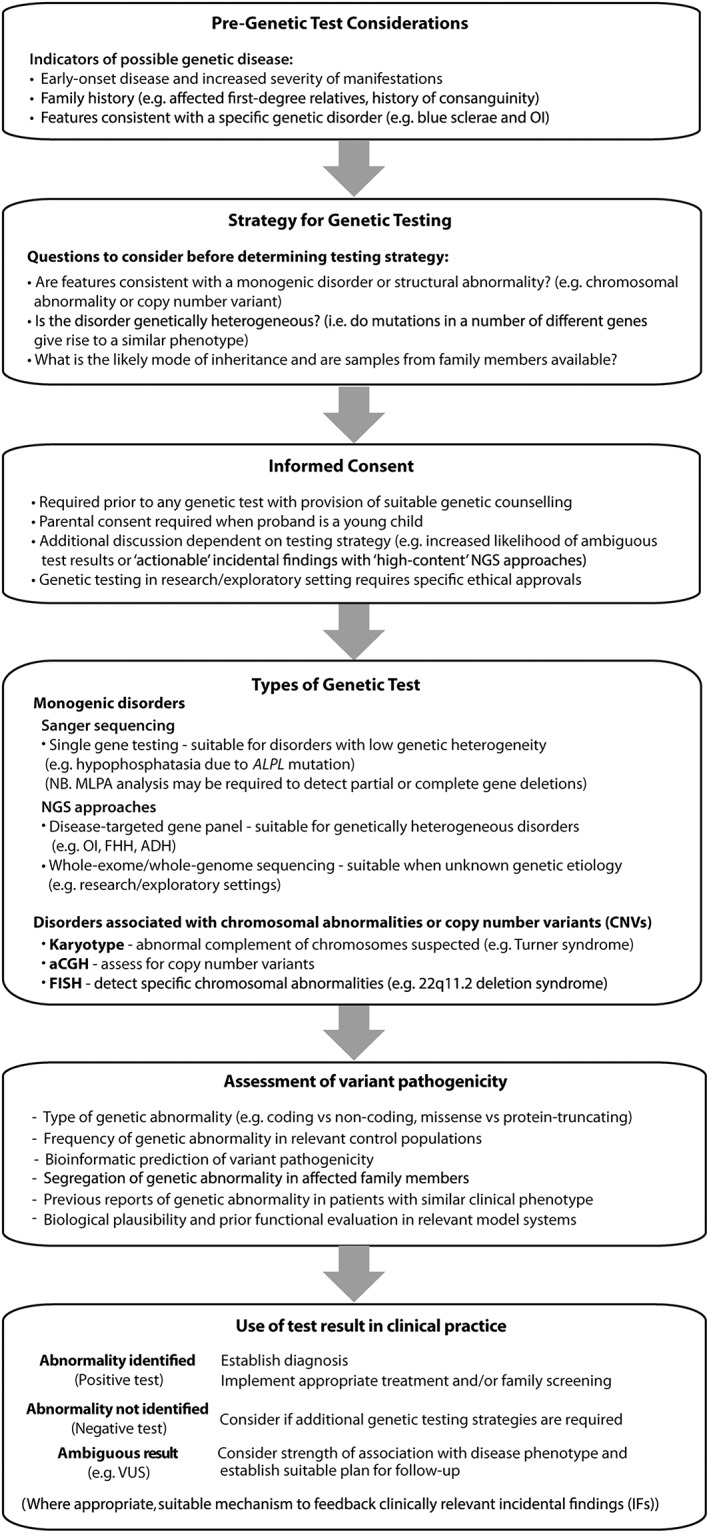
Flowchart outlining considerations for genetic testing in patients with metabolic bone disease

Establishing the genetic basis of a metabolic bone disease may aid diagnosis, treatment and prognostication; identify the need for screening of associated clinical features not initially apparent; enable appropriate genetic counselling and testing of first‐degree asymptomatic relatives; and facilitate preconception and/or prenatal genetic evaluation (Figure 2). Genetic testing may also aid risk profiling. For example, osteoporosis‐associated SNPs have been reported to predict fracture risk in patients taking bisphosphonates 71, and other studies have identified potential genetic markers of bisphosphonate‐induced osteonecrosis of the jaw 72.

For patients presenting with a likely genetic metabolic bone disease, several factors require consideration before organizing genetic testing (Figure 2). These include the phenotype of the patient, the likely mode of inheritance, the potential genetic aetiology [e.g., aneuploidy, copy number variation (CNV), or single gene defect], and availability of additional pedigree members (Figure 2). For example, DNA sequencing of ‘trios’ (i.e. both parents and the affected proband) may facilitate the identification of compound heterozygous or *de novo* mutations 73. Selecting the most appropriate genetic test will increase the likelihood of achieving a genetic diagnosis. For example, direct DNA sequencing methods which detect nucleotide abnormalities (e.g. substitutions, micro‐deletions and micro‐insertions) that cause most monogenic metabolic bone disorders frequently do not detect whole or partial gene deletions that are associated with some monogenic syndromes, and are also not optimal for identifying large chromosomal abnormalities (e.g. 22q11.2 microdeletion in DiGeorge syndrome), whose detection requires alternative approaches (Figure 2 and Table 2) 74. For other monogenic disorders, it is also important to consider analysis of a panel of genes if genetic heterogeneity is likely (e.g. in FHH or OI) 9, 20. Thus, it is important to emphasize that genetic testing which fails to identify an abnormality does not exclude a genetic disease, but rather may reflect: an alternative genetic aetiology to the one being tested; limitations of the employed genetic methodology (i.e. inadequate resolution or coverage); or incorrect assumptions regarding the clinical phenotype or mode of inheritance 3. As a consequence, it may be necessary to undertake sequential or simultaneous genetic tests to ensure a complete evaluation, although such testing may be limited by cost and local availability.

**Table 2:** Examples of genetic tests, their molecular resolution and utility

Genetic test	Resolution	Abnormalities detected	**Additional notes**
**Detection of chromosomal abnormalities including CNVs**			
**Karyotype: G‐banding (trypsin‐Giemsa staining)**	5–10 Mb	Aneuploidy	Limited resolution
		Large chromosomal deletions, duplications, translocations, inversions, insertions	Requirement to study many cells to detect mosaicism
**Fluorescence in situ hybridization (FISH)**	50 kb–2 Mb (dependent on size of probes employed)	Structural chromosomal abnormalities (e.g. microdeletions, translocations)	Labour‐intensive
			Low resolution limits its use
			Unsuitable where unknown genetic aetiology
**Multiplex‐ligation probe amplification**	Probe dependent	Copy number variations (CNVs) including (partial) gene deletions or duplications	Low cost, technically simple method
	50–70 nucleotides		Simultaneous evaluation of multiple genomic regions
	Single exon deletion or duplication possible		Not suitable for genome‐wide approaches
			Not suitable for analysis of single cells
**Array comparative genomic hybridization (aCGH)**	10 kb (high resolution)	Genome‐wide copy number variations (CNVs)	Inability to detect balanced translocations
	1 Mb (low resolution)		Useful for detection of low level mosaicism
	(Dependent on probes set)		
**Single nucleotide polymorphism (SNP) array**	~50–400 kb	Genome‐wide detection of SNP genotypes	Inability to detect balanced translocation
	(Dependent on probe set)	Copy Number Variations (CNVs)	Useful for detection of low level mosaicism
			Detection of copy number neutral regions or absence of heterozygosity (i.e. due to uniparental disomy)
**Detection of monogenic disorders (and CNVs)**			
***First generation sequencing (Sanger)***			
**Single gene test**	Single nucleotide	Single nucleotide variants (SNVs)	Relative high cost/base
	(exonic regions and intron/exon boundaries of candidate gene)	Small insertions or deletions (‘indels’)	May miss large deletions/duplications
			Unsuitable where unknown genetic aetiology
***Next generation sequencing***			
**Disease‐targeted gene panels**	Single nucleotide	Single nucleotide variants (SNVs)	May lack complete coverage of exomic regions (may require Sanger sequencing to fill in ‘gaps’)
	(exonic regions and intron/exon boundaries of candidate genes)	Small insertions or deletions (‘indels’)	Increased likelihood of identifying variants of uncertain significance (VUS) as number of genes increases
			Unsuitable where unknown genetic aetiology
**Whole exome sequencing (WES)**	Single nucleotide	Single nucleotide variants (SNVs)	Not all exons may be covered/captured
	(all exonic regions and intron/exon boundaries)	Small insertions or deletions (‘indels’)	Difficulties with GC‐rich regions and presence of homologous regions/pseudogenes
		Copy number variations (CNVs)	Small indels may not be captured
			Bioinformatic expertise required for data analysis
			High likelihood of incidental findings and VUSs
			Detection of CNVs requires additional data analysis (i.e. loss of heterozygosity mapping across exonic regions)
			Suitable for disease associated gene‐discovery
**Whole genome sequencing (WGS)**	Single nucleotide	Single nucleotide variants (SNVs)	Relative high cost
		Small insertions or deletions (‘indels’)	Large data sets generated and complex data analysis requiring bioinformatic expertise
		Copy Number Variations (CNVs)	High likelihood of incidental findings and VUSs
		(Translocations/rearrangements)	CNV analysis possible but may present specific challenges
			Suitable for disease associated gene‐discovery

CNVs, copy number variants; FISH, fluorescence in‐situ hybridization; LOH, loss of heterozygosity; WES, whole exome sequencing; WGS, whole genome sequencing. Adapted from Thakker, Whyte, Eisman, Igarashi, eds., *Genetics of Bone Biology and Skeletal Disease*, 2nd ed. Amsterdam: Academic Press, 2018: 14 3

### Types of genetic tests available to the clinician

#### Cytogenetic and molecular cytogenetic analyses

Karyotyping represents the initial test for major chromosomal abnormalities including aneuploidy or large insertions, deletions, duplications, inversions or reciprocal translocations, but has a resolution limited to ~5–10 Mb of DNA (Table 2) 74, 75. It retains an important place in the diagnosis of Turner and Klinefelter syndrome, each of which may manifest a form of osteoporosis 76, 77. Fluorescence *in‐situ* hybridization (FISH) employs DNA probes that hybridize to specific target regions, which allow the detection of specific chromosomal deletions, duplications, translocations or inversions (Table 2). The utility of FISH is limited to detecting abnormalities involving pre‐determined genomic regions (e.g. detection of 22q11.2 deletion in DiGeorge syndrome). Multiplex‐ligation dependent probe amplification (MLPA) detects complete or partial gene deletions by using a pool of custom‐designed probes to amplify specific genomic regions of interest (Table 2). MLPA is used in the diagnostic evaluation of monogenic disorders associated with such genetic alterations (e.g. MEN1) 78. Modifications of the MLPA technique may also be used. For example, in establishing the diagnosis of pseudohypoparathyroidism type 1b (PHP1b), methylation‐specific MLPA (MS‐MLPA) may be employed to detect genetic (e.g. deletions) or epigenetic (e.g. altered patterns of methylation) abnormalities within the differentially methylated regions (DMRs) of the *GNAS* locus, although alternate methods such as CpG bisulphite pyrosequencing are frequently used to confirm the presence of specific methylation defects 79. Microarray‐comparative genomic hybridization (aCGH) is undertaken for the genome‐wide detection of small chromosomal abnormalities (e.g. CNVs) (Table 2) and is increasingly used as a first‐line investigation for patients with multiple congenital abnormalities, which include skeletal manifestations and/or neurodevelopmental delay 80, 81. However, it is important to note that all individuals harbour many small CNVs without discernible adverse impact on health, whilst several potentially pathogenic CNVs do not cause disease in all individuals (i.e. reduced penetrance). Finally, SNP arrays may detect CNVs as well as facilitating genome‐wide genotyping (Table 2). For example, deletions spanning several adjacent SNPs included on the array may reveal loss of heterozygosity (LOH), whilst copy number gains (e.g. duplication) may be indicated by increased numbers of different genotypes 74. SNP arrays may also help localize recessive disorders in the offspring of consanguineous parents by facilitating homozygosity mapping 82, whilst regions of LOH can also indicate uniparental isodisomy, which may be relevant to the diagnosis of imprinting disorders such as PHP1b 83, 84.

### DNA sequence analysis

Sanger sequencing remains the gold standard for detecting DNA sequence variants due to the high accuracy of the DNA polymerase (i.e. base accuracy of >99.99%) employed during DNA amplification 41, 85. However, it remains labour intensive and is typically reserved for disorders with low genetic heterogeneity (e.g. single‐ or pauci‐gene disorders), an example being hypophosphatasia caused only by *TNSALP/ALPL* mutations 86. Single‐gene testing by Sanger sequencing is increasingly being replaced by next‐generation sequencing (NGS) approaches, which facilitates the simultaneous sequencing of large amounts of genetic material. Such NGS methodology has provided a paradigm shift in the investigation and diagnosis of genetic disease. Currently, the three most widely employed uses of NGS are whole genome sequencing (WGS), whole exome sequencing (WES), and disease‐targeted gene panel sequencing (Table 2). WGS determines the DNA sequence of the entire genome including coding and non‐coding regions, and can identify SNVs, small insertions or deletions (‘indels’) and CNVs 3. In contrast, WES analyses the 1–2% of the genome that encodes the ~20 000 protein‐coding genes (i.e. the ‘exome’), which are expected to harbour most disease‐associated mutations 3. WES has been the mainstay of highly successful disease‐gene discovery studies over the past decade, resulting in the identification of several genes responsible for metabolic bone disorders (e.g. *WNT1* mutations as causes of osteoporosis and OI 19; *SFRP4* mutations in Pyle's disease 54; AP2σ mutations in FHH type 3 33; *PLS3* mutations in X‐linked osteoporosis 18; *BMP1* mutations causing increased BMD and recurrent fractures 87; and *CYP3A4* mutations in vitamin D‐dependent rickets, type 3 88). Disease‐targeted sequencing represents the most widely utilized NGS method in clinical practice, as it can be designed to simultaneously analyse large collections of genes (e.g. <10 to >150 genes) associated with a specific disorder 41, 84, 89. Such NGS disease‐targeted panels have been established for genetically heterogeneous disorders including OI and other skeletal disorders, as well as for hypophosphataemic rickets and calcium‐sensing disorders 90, 91, 92.

### Genetic tests to detect mosaicism

Some metabolic bone disorders only manifest as somatic mosaicism (e.g. *GNAS* mutations in MAS) 67. However, other conditions (e.g. OI type II) may also rarely occur as germline mosaicism, arising from somatic mutation during gametogenesis, and may cause diagnostic confusion. In this setting, apparently unaffected parents (with one carrying the mutation limited to their gametes) may give rise to more than one affected child, suggesting possible autosomal recessive inheritance, in contrast to the underlying autosomal dominant inheritance pattern 93. Detection of mosaicism has been enhanced by improved genome‐wide testing strategies (e.g. aCGH, SNP arrays, droplet digital PCR and NGS approaches), which can provide sensitive methods for the detection of low‐level mosaicism (e.g. 5% for SNP array) 70, 94, 95. However, choosing the optimal test depends on the clinical phenotype, the type of mutation suspected (e.g. SNV, CNV, aneuploidy), the likely extent of mosaicism, and its tissue distribution. Typically, circulating lymphocyte DNA will suffice, but analysis of other affected tissues may be required (e.g. fibroblasts or bone) 96, 97.

### Genetic tests for prenatal diagnosis

Prenatal genetic testing may be undertaken at pre‐implantation or prenatal stages*,* and has been used to detect severe skeletal disorders such as perinatal lethal OI 98. Pre‐implantation genetic diagnosis (PGD) uses a single cell taken from the developing embryo several days after *in vitro* fertilization (IVF) to detect chromosomal abnormalities or single gene defects, thereby allowing selection of the unaffected embryos for implantation 99. In contrast, prenatal genetic testing is used once pregnancy is established to identify fetuses at risk of genetic disease 99. Typically, this involves invasive methods such as chorionic villous sampling (CVS) or amniocentesis to obtain cells for genetic evaluation 99. This may include karyotyping for the detection of aneuploidy, FISH or aCGH to identify smaller chromosomal abnormalities or DNA sequencing to identify single gene defects associated with monogenic disease. Recent progress in the detection of cell‐free circulating fetal DNA in the maternal circulation (e.g. after ~10 weeks gestation) now offers the potential for non‐invasive prenatal genetic diagnosis (NIPD) and/or testing (NIPT) 100. Thus, a maternal blood sample may allow screening for aneuploidy and fetal sex determination, which is important for X‐linked disorders, and may also be used to detect monogenic disorders; however, this is limited to paternally inherited mutations or those arising *de novo*, as the sample may contain maternal cell‐free DNA, and hence the detected abnormality cannot be reliably assigned to the fetus as the methodology cannot distinguish between fetal and ‘contaminating’ maternal DNA in the sample 100.

### Data interpretation and incidental findings

The advent of high‐content genetic testing employing NGS approaches has revolutionized the investigation and diagnosis of genetic disease. However, such approaches may also present clinical and ethical challenges 101. For example, the simultaneous sequencing of large numbers of genes (e.g. disease‐targeted gene panels, WES and WGS) inevitably identifies variants of uncertain significance (VUS), whose relevance to the clinical phenotype is ambiguous 102, 103. Indeed, the methods employed to assess variant effects are frequently imprecise leading to inaccurate interpretation, although the provision of recent large‐scale population level sequence databases facilitates improved estimates of variant pathogenicity and penetrance 104, 105. In addition, high‐content genetic testing may identify clinically relevant genetic abnormalities unrelated to the phenotype under investigation [i.e. incidental findings (IFs)] and these may have important health implications for the patient and their family. Hence, the possibility of identifying ambiguous or incidental results should be part of the informed consent prior to genetic testing (Figure 2).

## Conclusion

Many metabolic bone diseases have a genetic basis, which may be a germline single gene abnormality (i.e. a monogenic or Mendelian disorder), a somatic single gene defect (i.e. a post‐zygotic mosaic disorder), or involve several genetic variants (i.e. oligogenic or polygenic disorders). Recognition of these heritable disorders is clinically important, as it can facilitate relevant and timely investigation and treatment for the patients and families. Recent advances in genetics and DNA sequencing methods have resulted in new ways to detect genetic abnormalities. Therefore, it is increasingly important for the clinician to gain an appreciation of these complex genetic tests and to combine this with the fundamental skills of history taking and physical examination to ensure they are used for the benefit of patients.

### Nomenclature of targets and ligands

Key protein targets and ligands in this article are hyperlinked to corresponding entries in http://www.guidetopharmacology.org, the common portal for data from the IUPHAR/BPS Guide to PHARMACOLOGY 111, and are permanently archived in the Concise Guide to PHARMACOLOGY 2017/18 112, 113, 114, 115.

## Competing Interests

There are no competing interests to declare.

*The authors are supported by: Wellcome Trust Investigator Award (106995/Z/15/Z) (to R.V.T.); National Institute for Health Research (NIHR) Oxford Biomedical Research Centre Program (to R.V.T.); NIHR Senior Investigator Award (NF‐SI‐0514‐10091) (to R.V.T.); Scottish Senior Clinical Fellowship funded by the Chief Scientist Office (CSO)/NHS Research Scotland (NRS) and the University of Dundee [SCAF/15/01] (to P.J.N.); and Shriners Hospitals for Children (to M.P.W.)*.

## Contributors

F.M.H., P.J.N., M.P.W., and R.V.T wrote the manuscript. All authors approved the final version.
